# Supplementary material for: Timing of Systemic Steroids and Neurodevelopmental Outcomes in Infants < 29 Weeks Gestation
Source: Children (Basel). 2022 Nov 3;9(11):1687. doi: 10.3390/children9111687 (PMC9688446; doi:10.3390/children9111687)
Supplement: Supplementary file 1 [file children-09-01687-s001.zip › children-1990286-supplementary.pdf]

Supplementary Table S1: Comparison of the baseline characteristics and short-term morbidities in the infants with and without follow-up data

Variables*	Follow-up data (N = 6,200)	No follow-up data (N = 3,877)	p - value
Maternal age, mean (SD)**	31.2 (5.8)	30.2 (5.9)	0.12
Maternal hypertension, % (n/N)	16.2 (984/6058)	14.1 (530/3756)	0.005
Maternal diabetes, % (n/N)	9.3 (551/5918)	8.6 (320/3709)	0.25
Antenatal steroid use, % (n/N)	89.6 (5444/6076)	88.2 (3335/3780)	0.03
Cesarean delivery, % (n/N)	58.1 (3589/6181)	56.5 (2176/3855)	0.11
Singleton, % (n/N)	72.8 (4513/6198)	75.2 (2909/3871)	0.01
Gestational age at birth (weeks), median (IQR)	26 (25, 28)	27 (26, 28)	< 0.001
Gestational age group, % (n/N)<br>22-25 (weeks)<br>26-28 (weeks)	30.9 (1918/6200)<br>69.1 (4282/6200)	24.4 (945/3871)<br>75.6 (2926/3871)	< 0.001
Birth weight (g), mean (SD)	924 (236)	985 (255)	< 0.001
Sex (male), % (n/N)	53.8 (3331/6192)	54.3 (2101/3866)	0.59
Small gestational age, % (n/N)	8.5 (524/6195)	6.5 (251/3866)	0.003
Apgar score < 7 at 5 min, % (n/N)	39.3 (2412/6139)	38.8 (1480/3811)	0.65
SNAP-II score > 20, % (n/N)	28.3 (1744/6170)	21.1 (813/3849)	< 0.001
Severe neurological injury (≥ grade III IVH or PVL), % (n/N)	13.4 (812/6046)	11.9 (444/3727)	0.03
PDA, % (n/N)	58 (3574/6161)	51.8 (1996/3851)	<0.001

*Data are presented as mean (SD), median (IQR) or % (n/N) as appropriate
** IQR = inter-quartile range; IVH = intraventricular hemorrhage; N = number; PDA = Patent ductus arteriosus; PVL = Periventricular leucomalacia; SD = standard deviation; SNAP-II = Score for Neonatal Acute Physiology-II

Supplementary Figure S1: Relationship of the outcomes with the postnatal age of starting sPNS on unadjusted univariate analysis

A

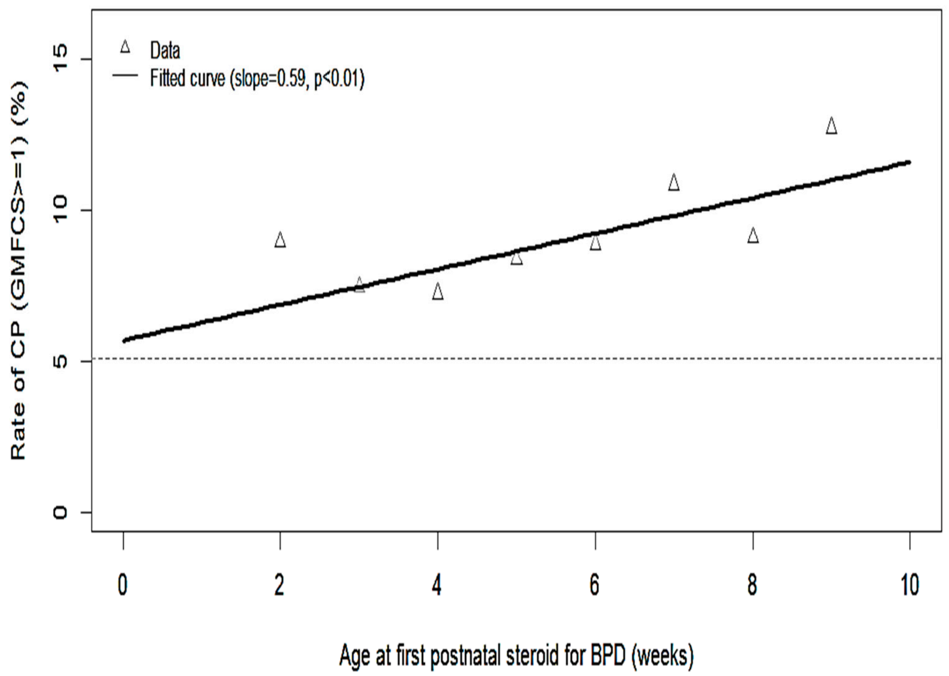

B

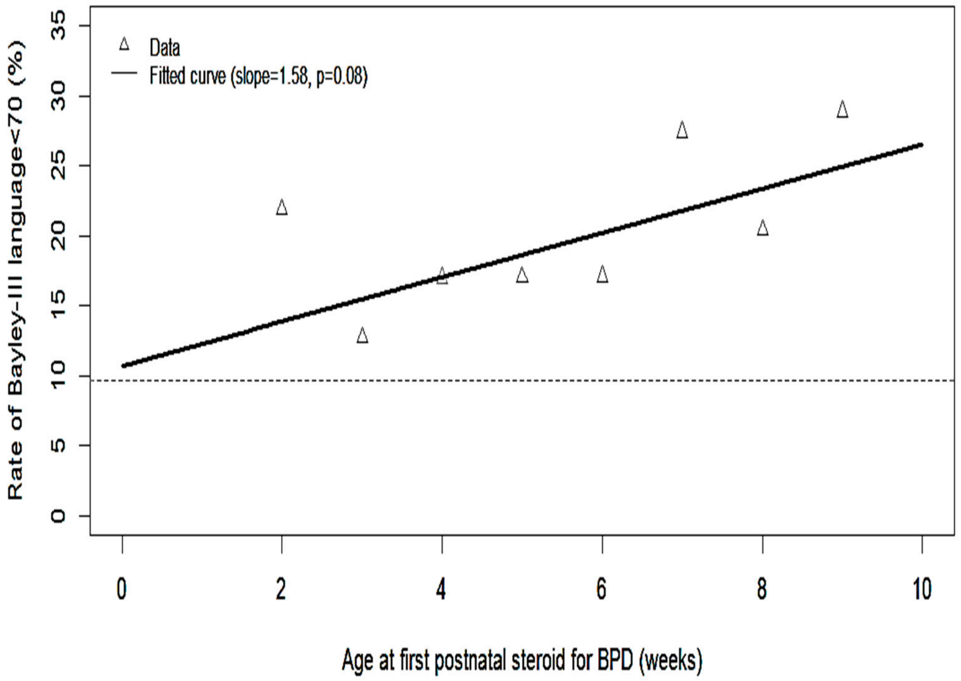

Data
Fitted curve (slope=1.58, p=0.08)
Rate of Bayley-III language<70 (%)
0
5
10
15
20
25
30
35
0
2
4
6
8
10
Age at first postnatal steroid for BPD (weeks)

C

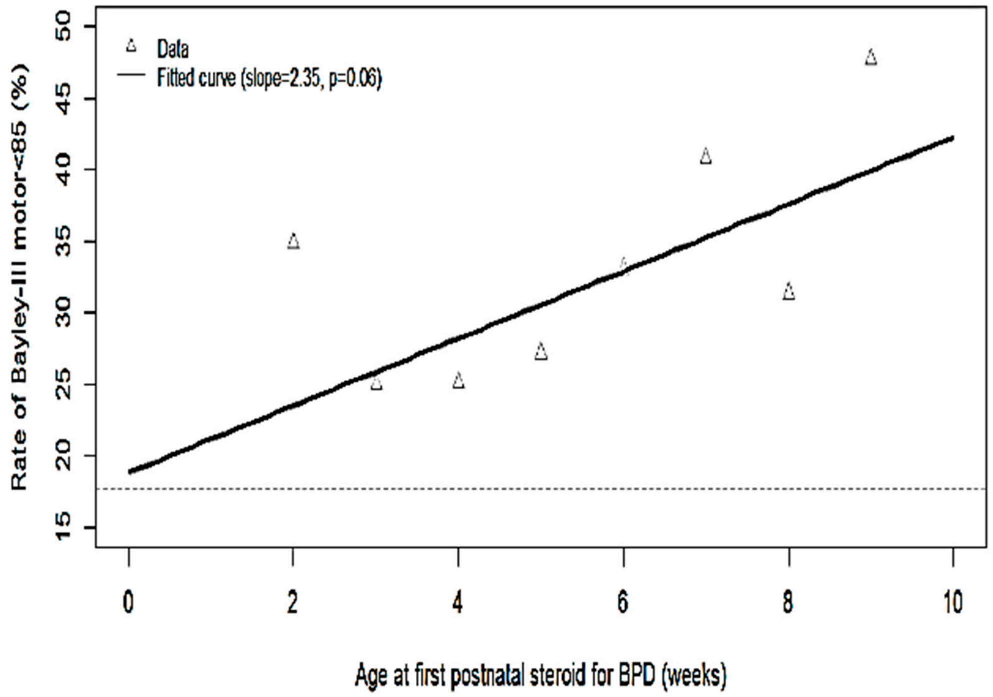

Data
Fitted curve (slope=2.35, p=0.06)
Rate of Bayley-III motor<85 (%)
15
20
25
30
35
40
45
50
0
2
4
6
8
10
Age at first postnatal steroid for BPD (weeks)

Supplementary Figure S2: Relationship of the outcomes with the postnatal age of starting of sPNS on unadjusted univariate analysis

A

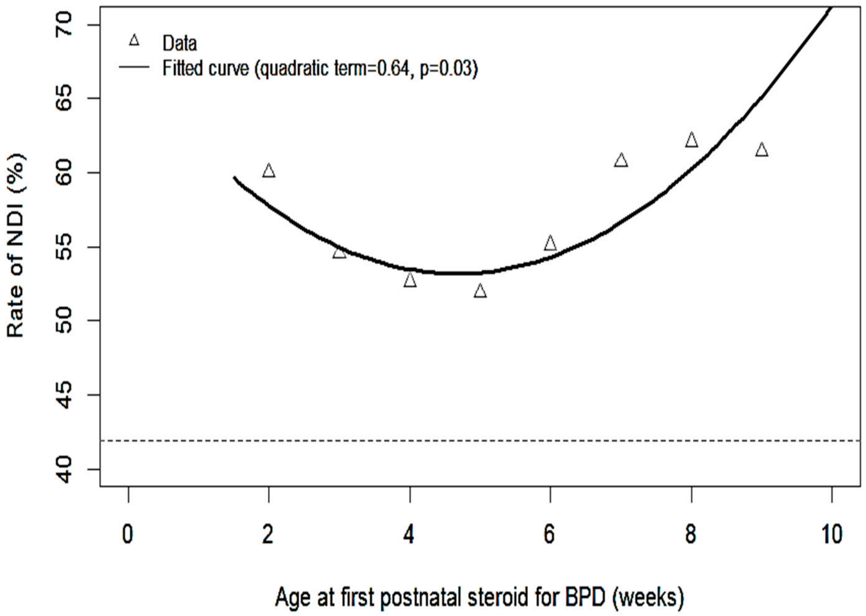

B

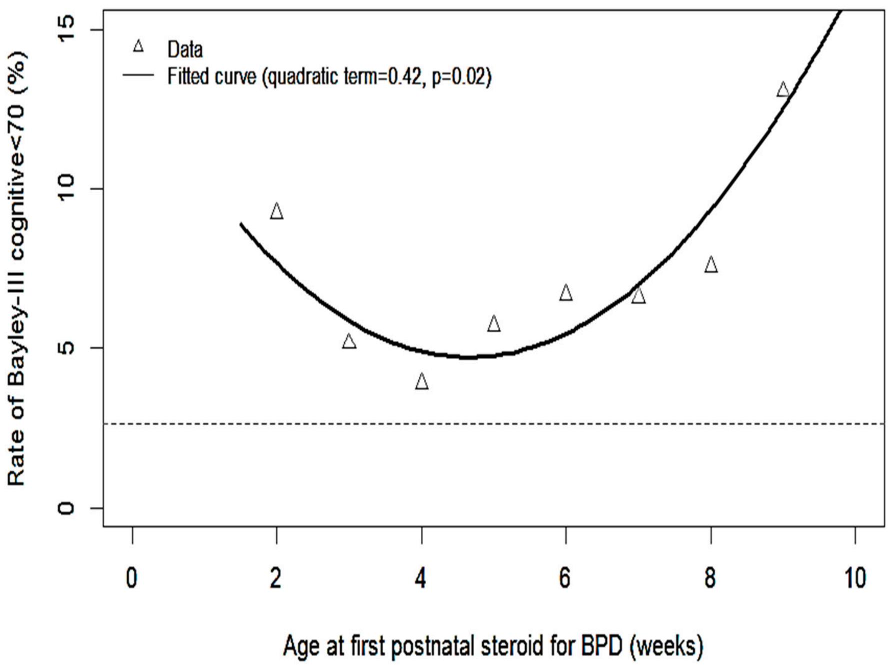

Data
Fitted curve (quadratic term=0.42, p=0.02)
Rate of Bayley-III cognitive<70 (%)
0
5
10
15
0
2
4
6
8
10
Age at first postnatal steroid for BPD (weeks)

C

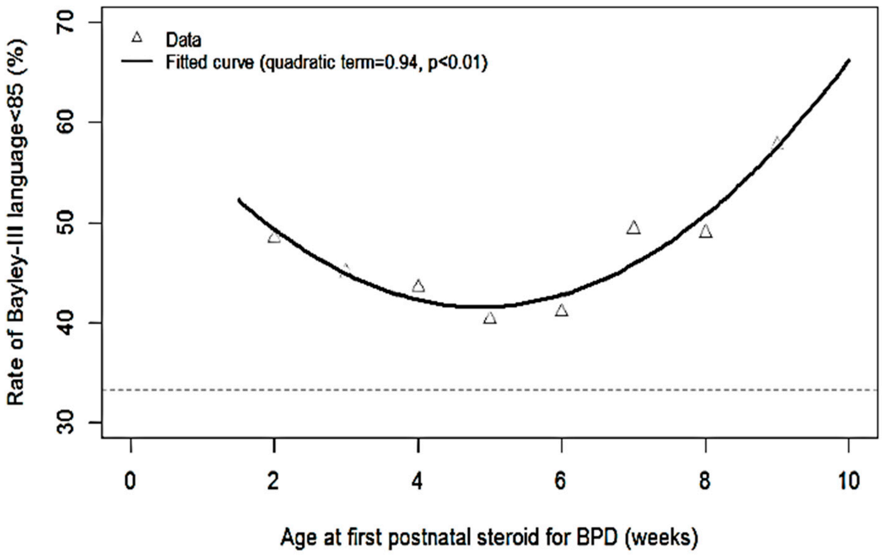

Data
Fitted curve (quadratic term=0.94, p<0.01)
Rate of Bayley-III language<85 (%)
30
40
50
60
70
0
2
4
6
8
10
Age at first postnatal steroid for BPD (weeks)

D

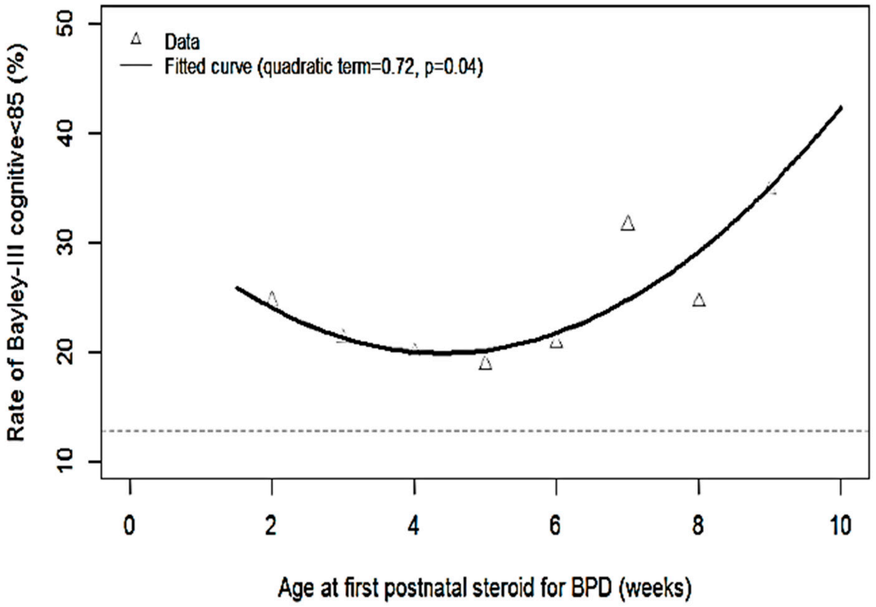
Data
Fitted curve (quadratic term=0.72, p=0.04)
Rate of Bayley-III cognitive<85 (%)
10
20
30
40
50
0
2
4
6
8
10
Age at first postnatal steroid for BPD (weeks)
